# Gut–Brain Axis and Perioperative Gut Microbiome in Postoperative Cognitive Dysfunction: Implications for Neurosurgical Patients

**DOI:** 10.3390/medsci13040236

**Published:** 2025-10-21

**Authors:** Ismail A. Abdullah, Sariya Khan, Fatma E. Hassan

**Affiliations:** 1 College of Medicine, Alfaisal University, Riyadh 11533, Saudi Arabia; 2 General Medicine Practice Program, Batterjee Medical College, Jeddah 21442, Saudi Arabia; sariyak2003@gmail.com; 3 General Medicine Practice Program, Department of Physiology, Batterjee Medical College, Jeddah 21442, Saudi Arabia; fatma.hassan@bmc.edu.sa; 4 Medical Physiology Department, Kasr Alainy, Faculty of Medicine, Cairo University, Giza 11562, Egypt

**Keywords:** gut-brain axis, perioperative, microbiome, neurosurgery, probiotics, postoperative neurocognitive disorders

## Abstract

**Background:** Postoperative cognitive dysfunction (POCD) is a common postoperative condition after neurosurgery, and in patients of advancing age, with far-reaching implications for recovery and quality of life. Current evidence points to the gut–brain axis as the main mechanism for the regulation of perioperative neuroinflammation and cognition. **Objective:** The aim of this review is to consolidate the existing evidence for perioperative gut microbiome dysbiosis in POCD, specifically in neurosurgical patients. **Methods:** A review of preclinical and clinical evidence on the gut microbiome, surgical stress, and cognitive recovery was conducted. Both mechanistic and therapeutic evidence were examined. **Results:** Surgery and anesthesia enhance gut microbial diversity, intestinal permeability, and systemic inflammation, thereby compromising neuroplasticity and the integrity of blood–brain barriers. Preclinical models show that interventions to reestablish microbial homeostasis with probiotics, prebiotics, or fecal microbiota transplantation decrease postoperative cognition. Clinical studies offer evidence supporting the associations between decreased short-chain fatty acid-producing bacteria and POCD risk. Randomized controlled trials have demonstrated that perioperative probiotics lower the incidence and markers of POCD. Multi-omic approaches to integrating microbiome, metabolome, and neuroimaging signatures are being engineered to discern recovery phenotypes prior to surgery. **Conclusions:** Perioperative gut microbiota are a modifiable target for the optimization of cognitive recovery from neurosurgery. The inclusion of microbiome treatments and diagnostics into standard surgical care pathways is one potential pathway to POCD minimization, but large randomized trials will be necessary to establish this.

## 1. Introduction

Postoperative cognitive dysfunction (POCD) is a recently realized postoperative condition, especially in old patients, which describes a new cognitive decline after recovery from anesthetic and surgical processes [1]. In total, 10–54% of the patients exhibit evidence of cognitive impairment a few weeks following significant surgery, with a higher incidence among old patients (~40% vs. 30% in young vs. old adults) [2,3,4]. Mild POCD also delays hospital stays and worsens functional recovery. Early postoperative deficit patients have augmented one-year long-term dementia and death [5]. Cognitive or dementia impairment, cerebrovascular disease, advanced age, and surgical stress degree are significant risk factors [3,4,5]. Though extremely prevalent, no pharmacologic therapy is effective on everyone; current perioperative therapy is aimed at the prevention of risks, like the maintenance of perfusion, and the prevention of delirium and supportive therapy [1].

Meanwhile, the gut–brain axis is a prime regulator of cognition. The gut microbiota harbors a dense microbial community whose metabolic and immune functions exert a significant impact on the host’s physiology [6]. Increasing evidence supports bidirectional interactions between gut microbes and the central nervous system (CNS) by neural (vagal), immune, and endocrine mechanisms. The gut microbiota also produces neuroactive compounds (e.g., serotonin, gamma-aminobutyric acid, and short-chain fatty acids (SCFAs)) that regulate the blood–brain barrier (BBB), microglia, and synaptic plasticity. But dysbiosis, or the loss of microbial diversity, provokes mucosal inflammation and generates neurotoxic metabolites like lipopolysaccharide (LPS) and trimethylamine-N-oxide that compromise the BBB and result in neuroinflammation [7]. Therefore, the microbiota can affect learning and memory significantly. A recent systematic review by Ramadan et al. [8] indicates in very high detail how gut microbiota composition and dynamics determine brain health and development across the life course, and how such influences are mediated by a bidirectional crosstalk via microbial metabolites, immune modulation, and neuroendocrine signaling. This adds to the growing evidence base proving that gut–brain interaction is not only necessary in the perinatal period but also responsible for enduring cognitive and affective resilience late into adulthood.

Even more significantly, surgical and anesthetic stressors cause derangement of this gut–brain axis. In mice, even brief isoflurane anesthesia strongly remodels gut microbiota, causing the microbiota to lose important commensal Clostridial [9]. More extensive surgery also induces gut dysbiosis: old mice undergoing laparotomy develop a microbial imbalance, increased intestinal permeability, and systemic metabolite disturbances, with an associated hippocampal neuroinflammation and synapse loss [9]. Some perioperative causes like anesthetic drugs, tissue trauma, pain, and antibiotics induce a transient dysbiosis and “leaky gut” [10]. The resultant inundation of circulating cytokines and microbial metabolites is a viable mechanism by which surgery induces neuroinflammation and interferes with postoperative neuroplasticity [7].

In addition to the explicit gut–brain axis, the gut–liver–brain axis also plays a significant role in neurocognitive well-being. As the central immunometabolic organ, the liver metabolizes gut-derived metabolites and endotoxins such as lipopolysaccharides and thereby modulates systemic inflammation and brain function. The hepatic stress caused by dysbiosis can also enhance neuroinflammation and prevent healing. New evidence indicates that disorders of this triad axis like alcohol use disorder, where liver disease and gut dysbiosis converge, cause cognitive impairment [11].

In this review, we first outlined the epidemiology and mechanistic models of POCD. We then reviewed the expanding role of the microbiota–gut–brain axis in neural plasticity and cognitive resilience, and reviewed evidence that perioperative insults like anesthesia, surgery, and others perturb this axis. We then looked at how gut microbes or their metabolites might be modulated perioperatively to create new opportunities for enhanced brain recovery from surgery.

## 2. Surgical Stress, Neuroinflammation, and Gut Dysbiosis

### 2.1. Endocrine–Immune Storm

**Figure 1:**
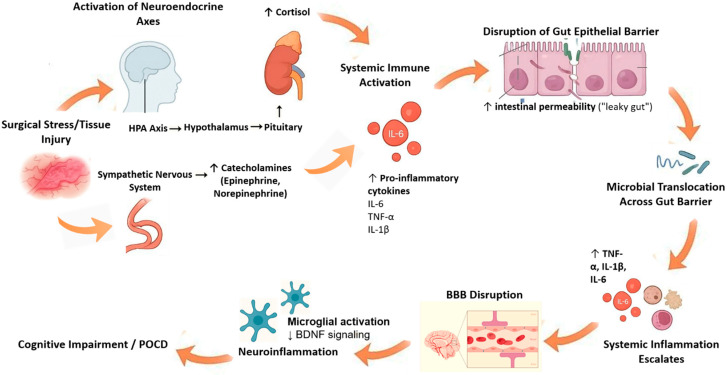
Endocrine–immune storm and its effects on the gut–brain axis. Figure Description: Surgical stress activates the HPA axis and sympathetic nervous system, causing a surge in cortisol and catecholamines. This endocrine response, together with elevated pro-inflammatory cytokines (e.g., interleukin [IL]-6, IL-1β, tumor necrosis factor [TNF]-α), disrupts intestinal epithelial tight junctions and promotes a “leaky gut.” Microbial products such as lipopolysaccharides then translocate into the bloodstream, amplifying systemic inflammation and impairing BBB integrity. The resulting neuroinflammation reduces BDNF signaling and contributes to POCD. Abbreviations: HPA: hypothalamic–pituitary–adrenal axis; IL: interleukin; TNF: tumor necrosis factor; BBB: blood–brain barrier; BDNF: brain-derived neurotrophic factor; POCD: postoperative cognitive dysfunction. ↑: increase, ↓: decrease.

Major surgical interventions trigger a profound neuroendocrine and inflammatory stress response. Afferent neural signals from injured tissue activate the hypothalamic–pituitary–adrenal (HPA) axis and sympathetic nervous system, resulting in a cortisol release and catecholamine surge [12,13]. Indeed, surgery markedly elevates systemic interleukin (IL)-6 and other cytokines. The magnitude of perioperative IL-6 rise correlates with tissue trauma severity and predicts poor outcomes [13]. Together with cortisol, these mediators orchestrate an “endocrine–immune storm.” Catecholamines and IL-6 can each disrupt gut epithelial tight junctions, promoting paracellular leak. For example, IL-6 upregulates the pore-forming junction protein claudin-2 via c-Jun N-terminal kinase (JNK)/activator protein-1 signaling, causing a size-selective increase in intestinal permeability [14]. In preclinical models, stress hormones have been shown to modify gut microbiota directly and activate mucosal immune cells (mast cells and macrophages), releasing proteases and vasoactive factors that perturb the epithelial barrier [15]. In summary, the perioperative cytokine and catecholamine surge both prime the intestinal barrier for breakdown and initiate a systemic inflammation that reaches the brain [14,15]. Figure 1 demonstrates this mechanism.

### 2.2. Gut Barrier Breakdown and Microbial Translocation

Concomitant with systemic inflammation, surgical stress compromises gut barrier integrity. Inflammatory mediators including IL-1β, tumor necrosis factor-α (TNF-α), and IL-6 and stress pathways like mast-cell degranulation and sympathetic ischemia increase epithelial permeability [15]. This so-called “leaky gut” enables luminal microbes and their products—LPS, peptidoglycan, flagellin, and many others—to translocate into the circulation. Multiple lines of evidence support this mechanism: for example, an animal study of laparotomy showed that the manipulation of the colon causes intraluminal LPS and labeled particles to penetrate the intestinal wall and migrate to the muscularis and blood [16]. Importantly, eliminating gut bacteria or deleting toll-like receptor (TLR) 4 (the LPS receptor) prevented the distant inflammatory response in this model, implying a key role for microbial TLR agonists in driving perioperative inflammation. Clinically, major operations have been associated with detectable endotoxemia and elevated serum endotoxin-binding protein postoperatively, consistent with translocation [17,18].

Once in the bloodstream, microbial debris potently activates innate immunity. LPS engages TLR4 on circulating monocytes and the vascular endothelium, triggering a cascade of TNF-α, IL-1β, and IL-6 release; peptidoglycan and lipoteichoic acid (from Gram-positive bacteria) act via TLR2 similarly. These signals amplify the systemic inflammatory response and can breach the BBB, promoting neuroinflammation and microglial activation. Thus, surgery-induced dysbiosis and barrier failure link peripheral inflammation to central inflammation. A recent mouse study crystallized this concept: aged surgical mice developed cognitive impairments concomitant with gut dysbiosis and increased gut permeability, whereas restoring a healthy microbiome or reinforcing the gut lining (via fecal microbiota transplantation (FMT), probiotics, glucocorticoids, or metabolite supplementation) ameliorated both barrier leak and brain inflammation [10]. In short, postoperative gut dysbiosis is both a consequence of surgical stress and an amplifier of the ensuing neuroinflammatory cascade [10,19].

### 2.3. Neurosurgery-Specific Factors

Neurosurgical patients face some unique exposures that may exacerbate the stress–dysbiosis cycle. Craniotomies and lengthy intracranial procedures often require prolonged general anesthesia and mechanical ventilation, intensifying neuroendocrine activation and hemodynamic fluctuations [20]. Although specific data are sparse, one may infer that an extended surgical duration magnifies the cumulative cortisol/catecholamine release and intestinal hypoperfusion, further compromising the gut barrier [21]. Moreover, neurosurgery entails dural opening and cerebrospinal fluid exposure; while aseptic, this could allow the bidirectional movement of immune mediators between the brain and periphery, although the net effect on gut–brain signaling remains to be studied [22]. Perioperative antibiotics are routinely administered in neurosurgery to prevent infection. Cefazolin or other cephalosporins (often given 24–48 h perioperatively) can temper systemic inflammation, but at a cost to the microbiome. In mice, perioperative cefazolin markedly reduced surgery-induced IL-6/TNF-α release in the brain and improved memory performance. However, cefazolin alone caused transient gut dysbiosis and colonic inflammation. Indeed, the same study showed that 5-day cefazolin after surgery completely prevented post-laparotomy memory deficits, yet mice given cefazolin without surgery exhibited subtle cognitive impairments, presumably from antibiotic-induced dysbiosis [18]. Clinically, high perioperative antibiotic use is known to drastically alter gut flora. Broad-spectrum agents indiscriminately kill commensals, allowing the overgrowth of opportunists [23]. Similar concerns apply to other neurosurgical medications (proton pump inhibitors (PPIs), opioids, immunosuppressants) and intensive care unit (ICU) interventions (parenteral nutrition, prolonged ICU stay), all of which have been linked to gut microbial shifts [24]. In summary, the typical perioperative regimen in brain surgery of prolonged anesthesia plus antibiotics and intensive care tends to amplify gut dysbiosis and barrier failure. This creates a feed-forward loop: dysbiosis sustains systemic inflammation, which in turn deepens neuroinflammation. In one review, stress hormones and perioperative interventions like antibiotics, opioids, and sterile gut prep, were specifically identified as factors that affect the relative abundance and diversity of the enteral microbiome after surgery [25].

Given this interplay, strategies that protect the gut microbiota or barrier may be especially beneficial in neurosurgical patients. For example, targeted probiotic/prebiotic therapy, perioperative SCFAs supplementation, or even selective antibiotic stewardship could theoretically mitigate POCD risk. These concepts remain experimental, but the mechanistic rationale is supported by data showing that reinforcing the intestinal barrier (e.g., with dexamethasone, tight-junction enhancers, or young-donor FMT) attenuates surgery-induced neuroinflammation and cognitive decline in animal models [10].

## 3. Mechanistic Links from Intestine to Brain

Gut microbes produce key metabolites that affect the BBB and plasticity. For example, germ-free or antibiotic-treated mice have reduced brain tight junction proteins, such as occludin and claudin-5, and a leaky BBB [26]. Colonizing germ-free mice with butyrate-producing strains (or giving a SCFAs mix) restores tight junctions and BBB integrity [26]. Butyrate (a histone deacetylase (HDAC) inhibitor) and related SCFAs also enhance neurotrophic signaling. Butyrate accelerates hippocampal brain-derived neurotrophic factor (BDNF) expression through chromatin relaxation and elevates BDNF levels in animal models [27,28]. Concurrently, SCFAs directly suppress microglial inflammation [29]. In vitro, acetate and butyrate each downregulate microglial cytokine release and nuclear factor kappa-B (NF-κB) activation after LPS stimulation [29]. Similarly, the tryptophan catabolite indole-3-propionic acid (IPA), generated by gut bacteria, is potently neuroprotective. Oral IPA reduced oxidative injury and microglial activation in ischemia models, and a human probiotic trial showed that raised IPA correlated with higher serum BDNF. In vitro, IPA lowered microglial TNF-α and boosted neuronal BDNF release [30,31]. Altogether, microbiome-derived SCFAs and indoles maintain BBB function, promote BDNF-driven plasticity, and keep microglia in a non-inflammatory state [26,27,28,29,30,31].

Gut signals also engage neuroimmune pathways. SCFAs in the gut lumen activate vagal afferents (via Free Fatty Acid Receptor 2/3) to relay information to brainstem centers (Nucleus of the Solitary Tract (NTS) and beyond) [29]. These vagal pathways project to loci such as the locus coeruleus and hippocampus, modulating arousal, learning, and memory in response to gut state [29]. Peripheral inflammation likewise influences brain immunity: circulating cytokines (e.g., IL-1β) can act on brain endothelial and choroid plexus receptors, altering neuroinflammatory tone [32]. Dysbiosis-driven endotoxin (LPS) and cytokines can cross a compromised BBB to activate glia. Thus, gut-derived IL-1β and other mediators provide an indirect route for enteric infection/inflammation to exacerbate central IL-1 signaling [33]. Interestingly, nutritional and drug interventions that enhance SCFA-producing bacteria even more strongly enhance such protective actions. Recent reviews recommend SCFA producers to be top microbial components for neuroprotection and cognitive resilience [34]. This reveals that the upkeep and drug enhancement of SCFA production might be an interventional treatable therapeutic modality.

Microbiome imbalance directly weakens barriers and primes glia. The resulting downregulated claudin-5 and occludin in the brain endothelium increases its permeability. In this dysbiotic state, barrier leakiness allows more microbial products (LPS) to enter circulation. At the same time, microglia become “primed” toward a pro-inflammatory (M1-like) phenotype. Germ-free or antibiotic-treated mice have immature, hyperactive microglia with exaggerated cytokine responses [26]. The absence of SCFA signaling removes a key anti-inflammatory check: normally SCFAs inhibit microglial NF-κB activity [29]. Recent work shows that antibiotic-induced dysbiosis triggers a microglial shift from M2 → M1, disrupting BDNF signaling and neurogenesis [35]. In summary, dysbiosis both compromises the BBB and biases microglia toward neuroinflammation.

Finally, perioperative drugs perturb the gut–brain axis. Volatile anesthetics (e.g., isoflurane) reduce gut microbial diversity, depleting beneficial Firmicutes and Lactobacilli and expanding Proteobacteria [36]. Propofol also shifts microbiome composition (e.g., lowering Prevotella and Lactobacillus) [36]. Opioids have even more profound effects: morphine rapidly alters gut flora within days, impairing the intestinal barrier and leading to systemic endotoxemia. Morphine-induced dysbiosis is associated with the loss of Bifidobacteria and Lactobacillaceae, and mice lacking a microbiome fail to develop opioid tolerance. These drug-induced microbiome changes likely amplify neuroinflammation (via LPS/leaky gut), compounding perioperative cognitive risks [37].

## 4. Preclinical Evidence Linking Gut Microbiota to Postoperative Cognitive Outcomes

Recent animal models support a causal role for the microbiome in perioperative brain outcomes. In aged mice undergoing surgery, anesthesia and tibial fracture induce gut dysbiosis and memory deficits. Jiang et al. [38] showed that anesthesia/surgery altered 37 gut genera and impaired reference memory; notably, either pre-surgery antibiotics or a probiotic cocktail restored 8 key bacterial taxa and entirely prevented the cognitive deficits [38]. Likewise, antibiotic-induced “pseudo–germ-free” mice exhibit exaggerated cognitive decline after surgery [39]. Xu et al. [39] found that such mice had abnormal postoperative behaviors that could be normalized by FMT from cognitively normal donors, whereas transplants from mice with postoperative delirium failed to help. These results indicate that a balanced gut microbiota is required for post-surgical cognitive recovery.

Similarly, gut–brain interactions influence other CNS injury models. In traumatic brain injury (TBI) models, probiotic intervention is protective. A recent study gave mice a multi-strain probiotic (including *Lactobacillus* spp. such as *L. rhamnosus*) before and after TBI. This treatment boosted fecal SCFA levels, reduced lesion volume and neuronal cell death, decreased microglial activation, and improved motor recovery (especially in males) [40]. In ischemic stroke models, enhancing SCFAs also aids recovery [41]. A systematic review and meta-analysis found that sodium butyrate treatment significantly raised hippocampal BDNF, lowered IL-1β and TNF-α in the brain, and reduced infarct volume and neurological deficits on sensorimotor and spatial tasks [29]. Notably, in a mouse model of post-stroke cognitive impairment, fecal transplants from cognitively impaired patients transferred deficits to stroke mice with elevated LPS and reduced butyrate, while oral butyrate supplementation rescued BBB integrity, reduced neuroinflammation, and improved cognition [35]. Thus, in stroke, as in POCD models, microbiota-derived LPS and a lack of SCFAs drive neuroinflammation and cognitive decline.

Spinal cord injury (SCI) models similarly illustrate this gut–CNS link. Spinal contusion causes gut dysbiosis, bacterial translocation, and worsened intraspinal inflammation. Importantly, feeding injured mice a multi-strain probiotic after SCI reduced spinal inflammatory cytokines and improved locomotor recovery. Conversely, antibiotic-induced dysbiosis impaired SCI recovery: antibiotic-treated or germ-free mice showed greater inflammation and worse functional outcomes, and these deficits could be partially reversed by microbiota reconstitution [42].

## 5. Clinical Evidence of Gut Microbiota Alterations and Cognitive Recovery in Neurosurgical and Perioperative Patients

### 5.1. Cross-Sectional Snapshots: Microbiota Signatures in Brain Tumor Patients

Based on a cross-sectional analysis of primary brain tumor patients, including patients with gliomas and meningiomas, the gut microbiota has demonstrated uniform dysbiosis patterns with respect to healthy controls. A pilot study comparing 32 patients with benign meningioma and 27 patients with malignant glioma (with 41 healthy volunteers) revealed a diminished α-diversity and altered microbial composition in both groups of tumors compared with control individuals. Meningioma patients showed a significant Enterobacteriaceae increase, whereas glioma patients showed no enrichment of Fusobacterium but enrichment of Akkermansia. Both tumor groups also showed a significant Firmicutes family decrease, in particular notable SCFA-producing members Lachnospiraceae and Ruminococcaceae [43].

These findings are corroborated by a larger meta-analysis of tumor-associated microbiota for brain tumors, which also reported concordant trends between the tumors: increased phyla Bacteroidetes, Proteobacteria, and Fusobacteria with decreased Firmicutes and Actinobacteria. Genus-level rises in higher Bacteroides, Prevotella, Phascolarctobacterium, and Escherichia/Shigella were observed, accompanied by decreases in Agathobacter, Lachnospira, and Parasutterella. These changes were manifested through an increased pro-inflammatory potential and the disruption of butyrate synthesis function in the tumor-associated microbiome [44].

In detail, glioblastoma multiforme (GBM) patients are found to have a lower microbial evenness and richness than healthy controls and a higher representation of Bacteroidetes and Fusobacteria and lower Firmicutes, Actinobacteria, and Verrucomicrobia. The changes lower the representation of protective taxa, i.e., Faecalibacterium and Akkermansia, associated with gut barrier integrity and anti-inflammatory activity [45].

Moreover, mechanistic orthotopic glioma mouse model experiments reinforce these human findings. In high-grade glioma microbiota-transplanted mice, taxa such as Eisenbergiella, Mailhella, and Merdimonas were enriched and associated with increased concentrations of tumor-supportive metabolites such as sphingosine-1-phosphate, while healthy donor microbiota enriched Limosilactobacillus and Anaerospora, which was associated with anti-inflammatory lipid metabolites [46].

Together, these cross-sectional findings reveal a convergent pattern in brain tumor patients: a decrease in SCFA-producing Firmicutes, a decreased gut microbiome diversity overall, and a new expansion of pro-inflammatory and possibly pathogenic taxa. Direct correlations to tumor-associated epilepsy severity are still empirically unproven in neurosurgical cohorts, though inflammatory mediators from the gut such as LPS and microbial metabolites provide a potential mechanistic link to enhanced neuroinflammatory susceptibility. These microbiological patterns concur with epilepsy-associated dysbiosis reported in more encompassing neuromicrobiome publications to propose a potentially biologically relevant axis amenable to further targeted investigation.

### 5.2. Emerging Evidence: Longitudinal

Although longitudinal neurosurgical patient microbiota experiments are rare, data are accumulating in the general POCD literature. In a preclinical study published in 2023, Wei et al. transplanted postoperative cognitive decline elderly surgical patients’ microbiota into antibiotic-treated rats. Recipients showed systemic inflammation and cognitive decline postoperatively (repressed TNF-α and IL-6), but nonrecipients receiving microbiota from non-POCD patients did not. This establishes a causality between baseline dysbiosis and postoperative brain dysfunction [47].

Clinical samples of elderly orthopedic patients also have preoperative microbial signatures with predictive POCD. Bi et al. [48] analyzed 40 older patients for 16S ribosomal ribonucleic acid (rRNA) and metabolomics and reported 20 bacterial genera and certain metabolites such as SCFAs to be significantly different between POCD and control patients. The markers were highly diagnostic in receiver operating characteristic (ROC) analysis. Both animal and human results highlight the significance of perioperative time-course dynamics of microbiota towards neurocognitive outcomes. Even though such reports present causal correlations among gut dysbiosis and POCD, the vast majority of human studies are confounded through restricted sample size and follow-up. Most of the designs in the literature are cross-sectional and hence do not allow temporal dynamics. Baseline confounders such as diet, antibiotics, and comorbidities also are not closely controlled. These exclusions exclude generalizability and highlight the need for large well-controlled longitudinal cohorts. Table 1 lists selected preclinical and clinical studies linking gut microbiota modulation to postoperative neurocognitive outcomes.

### 5.3. Control for Confounding Variables in Study Design

A strict control of the major confounders—drug intake, diet, bowel preparation, and eating habit—must be made while studying gut microbiota in neurosurgical patients.

PPIs, opioids, and perioperative drugs can profoundly disrupt gut ecology. Even though there is not much data in cohorts specific to neurosurgery, the general perioperative literature indicates that these drugs suppress beneficial taxa (e.g., Bifidobacterium and Faecalibacterium) and increase opportunistic groups such as Enterococcus, Streptococcus, and Proteobacteria [53].

Nutritional constituents are also crucial. Fiber-rich diets favor SCFA producers, which favor gut barrier function and anti-inflammatory signaling that are beneficial to brain recovery. While not yet investigated in neurosurgical patients, food habits, e.g., Mediterranean versus (vs.) rice diets, need to be recorded as important baseline modifiers [54].

Bowel prep, usually performed before cranial imaging or surgery, affects acute microbial loss and facilitates the rapid regrowth of fast-dividing opportunists. In the absence of uniformly standardized sampling time points with regard to prep, microbial results may be influenced. Steroid treatment for cerebral edema management will further alter gut communities, though neurosurgery-related data await investigation [55].

## 6. Modulation of the Perioperative Microbiome for Therapy

The growing appreciation for the gut–brain axis in neurorecovery has generated enthusiasm for microbiome-guided therapies as adjuncts to standard perioperative management. Probiotics, antibiotic stewardship, customized nutrition, and FMT are emerging in therapeutic interventions to enhance neurocognitive recovery through maintaining or restoring gut–brain homeostasis [56].

Several RCTs have demonstrated that perioperative probiotic supplementation is effective in reducing postoperative cognitive impairment in the elderly surgical group. In a double-blinded RCT of 120 older patients undergoing elective non-cardiac surgery, patients who received multi-strain probiotics from admission through discharge had less postoperative cognitive impairment (5.1% vs. 16.4% on placebo; *p* = 0.046) and greater decreases in plasma IL-6 and cortisol levels at postoperative day 5–7. Fecal microbiota composition at the genus level also changed more in the probiotic group (showing enhanced microbial resilience) [49].

Similarly, in a randomized trial among elderly patients undergoing hip or knee arthroplasty, perioperative probiotics reduced POCD incidence (26.7% vs. 56.9%; relative risk (RR) 0.47; *p* = 0.003), with benefits being limited to verbal memory domains. This illustrates both an overall POCD reduction and a domain-specific cognitive benefit [50].

Outside of the operating theater, probiotics have shown cognition-improving effects in community-dwelling older subjects: a 12-week multicenter placebo-controlled trial of Bifidobacterium bifidum BGN4 and B. longum BORI improved mental flexibility, mood, and serum BDNF, with microbiota composition changes (e.g., increased Clostridiales) [57].

These findings collectively affirm that pre-habilitation with probiotics or synbiotics, especially multi-strain preparations vs. SCFA-producing genera, imbues cognitive advantages through putative anti-inflammatory mechanisms, HPA axis normalization, the promotion of BDNF, and microbial stabilization. While no neurosurgery-specific antibiotic–microbiome literature exists, the broader literature highlights that broad-spectrum and prolonged antibiotics cause extensive disruptions of gut microbial diversity, especially the anaerobic SCFA-producing taxa, and prolong dysbiosis for months to years. Stewardship guidelines mandate a reduction in unnecessary antibiotic therapy, duration reduction, and avoidance of anti-anaerobic agents where possible [58]. Given antibiotics’ proven link with increased ICU-acquired infection and gut dysbiosis rates, targeted de-escalation strategies in neurosurgical prophylaxis (e.g., cefazolin vs. vancomycin choice, shortening of duration) may preserve microbial integrity and potentially limit postoperative neuroinflammation [58].

Although enteral feeding strategies have not been tested in neurosurgical POCD patients, there is widespread reporting of enteral nutrition with added fermentable fiber to enhance SCFA-producing bacteria such as Roseburia, Anaerostipes, and Blautia, with beneficial impacts on gut barrier function, systemic inflammation, and neuroprotection via butyrate-mediated HDAC inhibition and BDNF upregulation [59,60].

Preclinical evidence in aged mice under surgery shows that prebiotics (xylo-oligosaccharide) or feeding restores tight junction proteins (Zonula-occludens-1 (ZO-1)), preserves BBB integrity, and suppresses cognitive impairment, showing that dietary intervention during the postoperative period can have neuroprotective actions [61,62].

Early enteral nutrition through the use of fiber-containing formulas supplemented with prebiotics or synbiotics is thus a promising strategy for gut–brain axis homeostasis. Experimental research shows that FMT in aged mice with cognitive impairment increased microbiota diversity, restored tight junction proteins (ZO-1, claudin-1, occludin), decreased intestinal permeability, reduced pro-inflammatory cytokines (IL-1β, IL-6), increased anti-inflammatory IL-10, and improved behavioral and cognitive function [63].

Clinical human evidence for FMT in cognitive disorders is limited, despite systematic reviews and case series indicating that FMT and probiotics enhance cognitive function in neurological groups like Alzheimer’s disease and mild cognitive impairment [64]. Future-generation approaches including defined microbial consortia and postbiotic metabolites (e.g., butyrate, indole-3-propionic acid) are being explored, with targeted modulation without whole-FMT risks. But definitive trials in surgical populations are needed. They are shown in some randomized trials to decrease rates of postoperative cognitive dysfunction in elderly non-neurosurgical patients (i.e., hip/knee arthroplasty and other nonsurgical cardiosuppression), as observed in meta-analyses of probiotic research in Alzheimer disease, mild cognitive impairment, and Parkinson disease patients. They are useful to a moderate degree in reducing symptoms or cognitive improvement in the nonoperative setting [50].

But, to our best current knowledge, no large, high-quality randomized controlled trials in older-aged surgical patients with diagnosed Parkinson’s or Alzheimer’s disease have yet been published that quantify the effect of probiotics on postop duration of delirium or postop cognitive decline/improvement slopes [49,65]. The evidence is thus at present derived from biological plausibility and proof-of-concept efficacy for microbiome-directed therapy, but is still not safely extrapolatable to surgical AD/PD patients. Prior to pursuing such trials (cognitively enriched preoperative status and standardized delirium/cognition outcomes), advice to give routine probiotic treatment to shorten the duration of delirium or reverse postoperative cognitive impairment in surgical patients with AD or PD is premature. Apart from probiotics and nutritional treatment, pharmacologic and herbal treatments are increasingly indicated to improve cognition via gut microbiota modulation. For instance, microbiota-directed therapy and traditional Chinese herbal prescriptions have been reported to enhance microbial metabolite production, inhibit neuroinflammation, and improve post-stroke cognitive recovery [66]. Although these findings are beyond the domain of neurosurgery, they open the door to drug–microbiome interaction as a therapy in perioperative brain recovery. Though they are enthusiastic, therapeutic trials are flawed methodologically in their restriction. Most clinical trials of synbiotics or probiotics are composed of small, single-center, elderly orthopedic patients who are not candidates for neurosurgery. Incomparability related to heterogeneity of intervention (different strains, dosing, and timing) and short-term hospital discharge for follow-up precludes any long-term cognitive outcome measures. Mechanistic data are delivered by animal models but perhaps are not the same as human surgical candidates. Table 2 summarizes the various therapeutic options targeting the perioperative microbiome.

## 7. Multi-Omics and Precision-Recovery Framework

**Figure 2:**
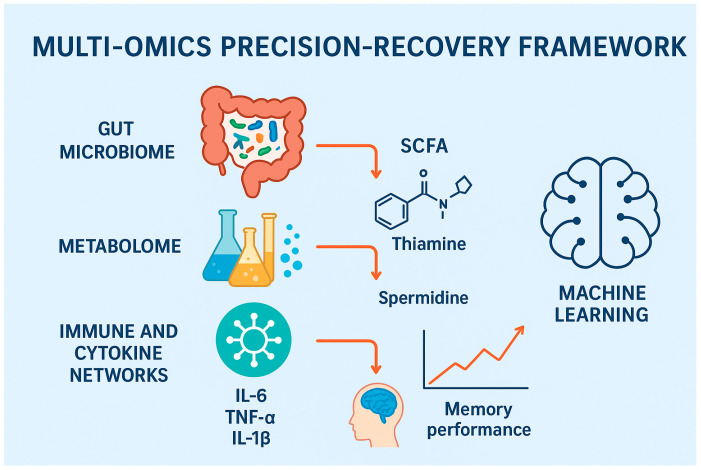
Multi-omics precision-recovery framework for perioperative cognitive dysfunction. Figure Description: This figure illustrates a very structured model with multiple levels of biology that affect cognitive function after surgery. At the bottom, the gut microbiota creates metabolites such as SCFAs and LPS, and dysbiosis leads to impaired homeostasis. The metabolome has downstream perturbation with deregulated SCFA, thiamine, and spermidine affecting cellular energy and neuroprotection. These metabolic derangements engage immune and cytokine networks such that elevated IL-6, TNF-α, and IL-1β cause systemic and neuroinflammation. The combination of these actions erodes brain function, measurable with the application of neuroimaging (e.g., MRI-based estimates of connectivity) and clinical examination of memory and attention. To the right is an integration module for machine learning that aggregates data from each layer to enable predictive modeling of POCD and patient-specific interventions for postoperative recovery.

To fully untangle the gut–brain axis role in perioperative cognitive outcomes, an integrated multi-omic approach is warranted. This entails shotgunning metagenomics, plasma metabolomics, cytokine profiles, and resting-state functional magnetic resonance imaging (rs-fMRI) to recognize multidimensional recovery phenotypes. These combined platforms allow for more intricate probing of biological pathways interlinking gut dysbiosis with disruptions in neural networks following surgery, as shown in Figure 2.

In a seminal preclinical investigation, Zhang et al. [71] conducted shotgun metagenomics on the fecal matter of aged POCD-model mice, which was typified by precipitous declines in butyrate-producing Lachnospiraceae and Eubacterium and increases in Bacteroides acidifaciens and Mucispirillum schaedleri. This was supplemented by untargeted plasma metabolomics, with a decrease in metabolites such as thiamine, spermidine, and long-chain unsaturated fatty acids markers of microbial metabolic derangement. Critically, these microbial and metabolic alterations were also reflected with behavioral deficits and neuroinflammation, linking peripheral multi-omic profiles to cognitive phenotypes.

Translated to humans, Liang et al. [72] performed an integrative analysis in patients with age-related cognitive impairment by combining 16S rRNA sequencing, targeted serum metabolomics, cytokine quantification, and structural MRI. They reported that certain genera Odoribacter, Butyricimonas, and Bacteroides were positively associated with hippocampal volume and were mediated by acetic acid concentrations. This indicates that the brain structural maintenance pontine micturition center is mediated by gut-derived metabolites.

Machine learning classifiers that are trained on stratified inputs are also highly predictive. In the POCD mouse cohort of Zhang et al., for example, a taxa Lachnospiraceae, Bacteroides, and Mucispirillum random-forest classifier and plasma TNF-α detected cognitive impairment with ~82% cross-validation accuracy [71]. Similarly, Liang et al.’s human sample employed LASSO regression, a comparable method to uncover microbial-metabolite clusters that are predictive of hippocampal volume and cognition, suggesting that models based on data can disentangle multifactorial associations [72].

To enable discovery and validation beyond the immediate research group, FAIR (Findable, Accessible, Interoperable, Reusable) repository implementation is necessary. New infrastructure, e.g., EMBL-EBI, NIH Metabolomics Workbench, and OpenNeuro, enables rich upload of omics data and metadata, e.g., shotgun sequences from stool, plasma metabolite panels, cytokine panels, neuroimaging scans, and relevant clinical variables like type of surgery, drugs, diet, and cognitive scores [73,74]. These repositories support federated analysis, meta-cohorts, and model tuning.

In addition to microbiome profiling, both transcriptomic and metabolomic analysis provide more precise data on cognitive consequences in the perioperative period. Metabolic alterations, such as SCFA, thiamine, and spermidine decreases, indicate microbial dysfunction and are related to synaptic plasticity and neuroinflammation. Transcriptomic analysis also reveals gene-expression alterations within immune and neurotrophic pathways that are related to postoperative brain recovery. Together, these multi-omic approaches highlight the necessity of integrating microbiome, metabolome, and transcriptome information to understand the gut–brain axis comprehensively throughout the perioperative setting [71,72,73,74].

## 8. Future Directions

Surgical stress profoundly disrupts the gut microbiome through processes like fasting, anesthesia exposure, antibiotic treatment, and opioid treatment. One study by Serbanescu et al. demonstrated that even a local perioperative exposure model in mice will cause long-term reductions in commensals like Lactobacillus, Roseburia, and Ruminococcus [75]. Human musculoskeletal surgery cohorts recognize a requirement for the harmonization of windows of microbiome sampling and stool and plasma sampling at standardized perioperative time points (e.g., 48 h pre-, POD1, POD7, POD30) [76]. Sequencing methodologies also need to be standardized: 16S amplicon analyses are less suitable in comparison with shotgun metagenomic sequencing with the capability of strain-level and functional gene resolution. Reviews of orthopedic microbiome studies highlight the benefit of hybrid sequencing methods in supporting data validity and comparability [75,76,77].

Dysbiosis inconsistencies with outcomes require the clinical trial testing of microbiome-directed interventions. Concrete evidence has been found in non-neurosurgical surgical populations, i.e., hip/knee arthroplasty patients where perioperative probiotics reduced POCD occurrence (26.7% vs. 56.9%), with simultaneous reductions in IL-6 and IL-1β [49]. Additional RCTs in elderly orthopedic patients repeat these reductions and augmented postoperative BDNF [77]. These findings form the foundation for the development of biomarker-driven enrichment trials in neurosurgical patients recruiting subjects with preoperative dysbiosis (e.g., reduced SCFA producers or reduced alpha diversity) for treatment with probiotics or synbiotics. Trials need mechanistic endpoints such as stool metagenomics, plasma SCFAs, cytokine patterns, gut barrier markers, and neurocognitive function to determine the biological effect of the intervention.

Long perioperative medications have a significant impact on the gut microbiota. Isoflavane anesthesia and prolonged fasting are sufficient to decrease the diversity of beneficial taxa in mice, as demonstrated by Serbanescu et al. [75]. The stewardship of antibiotics is required, since broad-spectrum medications drain SCFA producers for extended durations. Opioids and corticosteroids also disrupt gut barrier integrity. Conversely, nutrients and microbial metabolite-derived products can regulate drug metabolism, opioid clearance, and the breakdown of anesthetics. Systemically, subsequent studies must determine the effect of drug regimens on gut microbiota, and if the co-administration of microbiome-sparing compounds erases adverse effects.

The microbial community is strongly affected by diet variety, cleanliness, and antibiotic use issues that geographically vary. Current neurosurgical microbiome research tends to be biased toward high-income settings, and Westernized diets and may have limited generalizability elsewhere. Orthopedic microbiome reviews suggest the inclusion of low- and middle-income country (LMIC) participants, where diets are usually rice-based or fermented food-based [76]. Enrollment of these groups will unveil culture-specific microbiome recovery therapies, promote generalizability, and maximize research equity.

### Recommendations

We recommend the performance of a multicenter RCT involving those undergoing elective neurosurgery, with a special focus on those at risk for postoperative cognitive dysfunction, like elderly patients, with stratification based on initial gut microbiota profile status, i.e., the presence or absence of SCFA-producing taxa proven to be linked with cognitive resilience. Perioperative multi-strain synbiotics would be administered to the intervention arm and placebo to the control arm. The primary outcomes to be assessed will include functional neurocognitive tests, while the secondary outcomes will include the assessment of gut-brain interactions by stool metagenomics, plasma SCFA concentrations, inflammatory cytokines, markers of gut barrier integrity, and resting-state fMRI. Also, probable confounders such as dietary consumption, antibiotic exposure, and concomitant medications have to be stringently controlled. Heterogeneous populations from different geographic locations must be included in order to create external validity, and all data created by omics research must be deposited in FAIR-compliant databases to ensure transparency, reproducibility, and collaborative progress in the field.

## 9. Conclusions

The gut microbiota mediates perioperative brain recovery by modulating neuroinflammation, synaptic plasticity, and BBB function. Preclinical models demonstrate that surgical dysbiosis compromises cognition and is reversible through the normalization of the microbial community. Clinical trials, although restricted, associate microbiota profiles with a risk of POCD and demonstrate a promising early optimism regarding probiotics and prebiotics for improving outcomes. Multi-omic approaches are transforming recovery phenotypes and may be used to underpin microbiome-driven interventions. The rehabilitation of these discoveries in neurosurgical practice can be a game-changer, especially for high-risk patients. Rigorous trials and mechanistic validation are, however, necessary prior to widespread clinical use.

## Figures and Tables

**Table 1:** Summary of some experimental and clinical trials linking gut microbiota to neurosurgical outcomes.

Study	Model/Population	Intervention/Exposure	Key Microbiota Findings	Neurocognitive Outcomes
Wang et al. (2021) [49]	(Human) Elderly patients undergoing non-cardiac surgery (RCT)	Daily multi-strain probiotic pre- and postop vs. placebo	Enhanced microbial stability in the probiotic group	Reduced incidence of POCD at discharge compared to placebo
Hu et al. (2023) [50]	(Human) Elderly patients after hip/knee arthroplasty (RCT)	Probiotic supplementation perioperatively	Microbial modulation “Increased *Faecalibacterium* and *Bifidobacterium*”	Attenuation of POCD incidence vs. placebo
Wei et al. (2024) [47]	(Animal) Aged rats administered FMT from preoperative POCD patients vs. control donors	FMT into antibiotic-depleted rats	Recipients of POCD-patient microbiota had increased Desulfobacterota genera; altered β-diversity	Impaired spatial memory (Morris water maze), elevated serum/hippocampal TNF-α, IL-1β; increased microglial activation
Cheng et al. (2024) [51]	(Animal) Aged mice undergoing surgery (exploratory laparotomy)	Surgery vs. sham; some groups received FMT from young donors	Surgery induced gut dysbiosis: ↑ Bacteroides, ↓ Akkermansia	Aged surgery group developed cognitive deficits; FMT from young donors improved cognition, reduced microglial activation, and restored barrier function
Han et al., 2020 [52]	(Animal) APP/PS1 transgenic mice with surgical stimulation	XOS prebiotic supplementation	XOS restored tight junction proteins (ZO-1, occludin), improved microbial composition	Preservation of BBB integrity, reduced neuroinflammation, reversal of POCD-associated cognitive decline

Abbreviations: RCT: randomized control trial; POCD: postoperative neurocognitive dysfunction; FMT: fecal microbiota transplant; TNF-α: tumor necrosis factor alpha; IL-1β: interleukin 1 beta; APP/PS1: amyloid precursor protein/presenilin 1; XOS: xylo-oligosaccharide; ZO-1: Zona Occludens-1; BBB: blood–brain barrier, ↑: increase, ↓: decrease.

**Table 2:** Therapeutic strategies targeting the perioperative microbiome.

Study	Intervention	Evidence Base	Microbiota Effects	Neurocognitive/Clinical Outcomes
Hu et al. (2023) [50]	Perioperative probiotics	RCT in elderly non-cardiac surgery (hip/knee arthroplasty) in China	Modulated gut microbial composition (genus-level changes), likely improved resilience	Reduced POCD incidence (26.7% vs. 56.9%); better verbal memory; lowered IL-6 and IL-1β
Wang et al. (2021) [49]	Probiotic capsules (multi-strain)	Randomized double-blind trial in elderly non-cardiac surgery patients	Greater perioperative stability in gut genera; presumed SCFA-producer preservation	Lower postoperative cognitive impairment rate (6.3% vs. 22.2%); reduced IL-6 and cortisol
Yang et al. (2018) [67]	Prebiotic supplementation (B-GOS)	Rat model of abdominal surgery under isoflurane	Enriched beneficial taxa; mitigated microglial activation markers (Iba-1), M1/M2 balance preserved	Reduced surgery-induced cognitive impairment (novel object recognition task outcomes)
Han et al. (2020) [52]	XOS prebiotic	APP/PS1 transgenic mice with surgical stress	Restored microbial SCFA-producer taxa; preserved tight junction protein expression	Attenuated spatial memory deficits post-surgery (Morris water maze performance)
Parker et al. (2022) [68]	FMT—aged → young mice	Aged mice receiving young donor FMT intervention	Increased diversity; decreased pro-inflammatory taxa; improved tight-junction proteins	Reduced microglial activation; partial cognitive improvements in memory tests
Elangovan et al. (2022) [69]	FMT in Alzheimer’s mouse model	5xFAD mice receiving FMT from young wildtype mice	Shift toward healthy microbial profile; improved SCFA-associated metabolites	Significant cognitive improvement on Y-maze and novel object recognition; reduced amyloid burden
Long et al. (2024) [70]	Antibiotic stewardship	Review of gut microbiome recovery after perioperative antibiotic exposure	Avoidance of broad-spectrum antibiotics preserves anaerobic SCFA producers	Mitigates long-term dysbiosis, potential reduction in neuroinflammation risk

Abbreviations: RCT: randomized control trial; FMT: fetal microbiota transplant; POCD: postoperative neurocognitive dysfunction; SCFA: short-chain fatty acid; IL-6: interleukin 6; B-GOS: β-galacto-oligosaccharides; Iba-1: ionized calcium-binding adaptor molecule 1; M1/M2: microglial activation states; XOS: xylo-oligosaccharide; APP/PS1: amyloid precursor protein/presenilin 1; FAD: Familial Alzheimer’s disease.

## Data Availability

No new data were created or analyzed in this study.
